# No significant improvement in neuromuscular proprioception and increased reliance on visual compensation 6 months after ACL reconstruction

**DOI:** 10.1186/s40634-021-00338-x

**Published:** 2021-03-06

**Authors:** Frank Wein, Laetitia Peultier-Celli, Floris van Rooij, Mo Saffarini, Philippe Perrin

**Affiliations:** 1 Centre, Artics, Clinique Louis Pasteur, Nancy, France; 2 Faculty of Medicine and UFR STAPS, University of Lorraine, EA 3450, Development, Adaptation and Handicap, Villers-lès-Nancy, France; 3 ReSurg SA, Rue Saint-Jean 22, 1260 Nyon, Switzerland; 4 Laboratory for the Analysis of Posture, Equilibrium and Motor Function (LAPEM), University Hospital of Nancy, Vandoeuvre-lès-Nancy, France

**Keywords:** ACLR, Posturography, Proprioception, Postural control, Rehabilitation, Clinical evaluation

## Abstract

**Purpose:**

To determine the contributions of proprioceptive and visual feedbacks for postural control at 6 months following ACLR, and to determine their associations with knee laxity, isokinetic tests and clinical scores.

**Study design:**

Level IV, Case series.

**Methods:**

Fifty volunteers who received ACLR between May 2015 and January 2017 were prospectively enrolled, and at 6 months following ACLR, postural stability was assessed. Somatosensory ratios (somatic proprioception), and visual ratios (visual compensation), were calculated to evaluate the use of sensory inputs for postural control. Univariable regression analyses were performed to determine associations of somatosensory and visual ratios with knee laxity, isokinetic tests and clinical scores.

**Results:**

At 6 months following ACLR, the somatosensory ratio did not change, while the visual ratio decreased significantly from 5.73 ± 4.13 to 3.07 ± 1.96 (*p* = 0.002), indicating greater reliance on visual cues to maintain balance. Univariable analyses revealed that the somatosensory ratio was significantly lower for patients who performed aquatic therapy (β = -0.50; *p* = 0.045), but was not associated with knee laxity, muscle strength or clinical scores. An increased visual ratio was associated with patients who received hamstrings tendon autografts (β = 1.32; *p* = 0.049), but was not associated with knee laxity, muscle strength or clinical scores.

**Conclusion:**

At 6 months following ACLR, visual ratios decreased significantly, while somatosensory ratios did not change. This may suggest that there is little or no improvement in neuromuscular proprioception and therefore greater reliance on visual cues to maintain balance. The clinical relevance of this study is that posturography can provide useful information to help research following ACLR and to predict successful return to play.

## Introduction

Postoperative rehabilitation strategies and timing of return to play (RTP) are crucial to avoid graft failure following anterior cruciate ligament reconstruction (ACLR) [11, 15, 21, 24, 25, 36]. For this reason, residual laxity is sometimes assessed at different timepoints, to help clinicians finetune rehabilitation and RTP [13, 22, 28, 29, 32], although anterior tibial translation (ATT) measurements do not reflect conditions during physical activities [20].

Knee proprioception, which contributes to dynamic stability, is often impaired in ACL-deficient knees [19, 23, 26, 27], but starts to recover in the first 6 months following ACLR [3, 33]. Impaired knee proprioception can increase the risk of ACL re-tears [15, 24], as it affects both voluntary and involuntary movements [10] as well as dynamic balance [11, 14, 20]. Methods previously used to assess knee proprioception seldom provide objective and accurate measurements to detect subtle impairments, because they are performed without weight-bearing [6] or vision deprivation [8].

Reliable analysis of postural control can be achieved with modern posturography platforms [25], which can be combined with vision deprivation methods to determine the contributions of proprioceptive and visual feedbacks [8]. The utility of such devices has not yet been demonstrated for research on ACLR. The purpose of this study was therefore to determine the contributions of proprioceptive and visual feedbacks for postural control at 6 months following ACLR, and to determine their associations with knee laxity, isokinetic tests and clinical scores. The hypothesis was that proprioceptive feedback improves at 6 months after ACLR, irrespective of knee laxity, muscle strength and clinical scores.

## Methods

The authors prospectively enrolled 50 volunteers who received ACLR, between May 2015 and January 2017, by the senior surgeon (FW). All participants were amateur or professional athletes and provided written informed consent for participation in the study, which was approved by the National Ethics Committee (C.H.U. de Montpellier, # 2019_IRB-MTP_03-09) and registered at ClinicalTrials.gov (NCT02225613). Exclusion criteria were: (i) history of hypertension or neurological disease, (ii) use of psychotropic medication, or (iii) concomitant lower limb injuries that could interfere with postural control in the previous 3 months.

### Surgical technique

All patients were operated under general anaesthesia, in the supine position, using a tourniquet. The femoral tunnels (10 mm diameter) were drilled blind-ended, from the inside-out, using the anteromedial portal. Twenty-six patients (58%) received bone-patellar-tendon-bone (BTB) autografts, and 19 patients (42%) received hamstring tendon (HT) autografts fixed with femoral endobuttons and tibial screws (Smith and Nephew, Andover, MA). Patients returning to non-contact sports with predominant quadriceps involvement received HT grafts, while patients returning to contact sports received BTB grafts. Meniscal repair or meniscectomy were performed in 16 medial (36%) and 4 lateral (9%) compartments, and extra-articular tenodesis was performed in 22 knees (49%) that had high-grade pivot-shift.

### Clinical assessment

All patients were evaluated clinically before surgery and at a minimum follow-up of 6 months using the Lysholm score [34], International Knee Documentation Committee score (IKDC) [16], and Knee injury and Osteoarthritis Outcome Score (KOOS) [31]. Absolute and side-to-side difference of anteroposterior laxity were recorded using the GnRB device (Genourob, Laval, France), at a force of 200 N [7, 18]. Isokinetic testing was performed at 180°/s during 3 repetitions to assess muscle strength deficit at peak torque between the ipsilateral and contralateral leg [17].

### Postoperative rehabilitation

Patients were randomized to follow a conventional rehabilitation protocol, with or without additional aquatic therapy, 5 times per week, for a period of 3 weeks. In the first phase, the goal was to obtain full extension and 90° of flexion, and at 6 weeks, 120° of flexion. Partial weight-bearing (50% body weight) was allowed during the first 3 postoperative weeks if the preoperative static anterior tibial translation (ATT) was < 5 mm [13] and progressive full weight-bearing was allowed between 3 and 6 weeks. At 6 to 12 weeks, the goal was to get full range of motion, increase muscular strength and knee stability. At the end of this phase, the patient was expected to walk quickly and climb stairs. At 3–6 months, the goal was to regain the normal muscle strength and prepare for gradual return to sport. This phase consisted of heavy resistance strength training and exercises depending on type of sport practiced.

### Posturography measurements

**Fig. 1:**
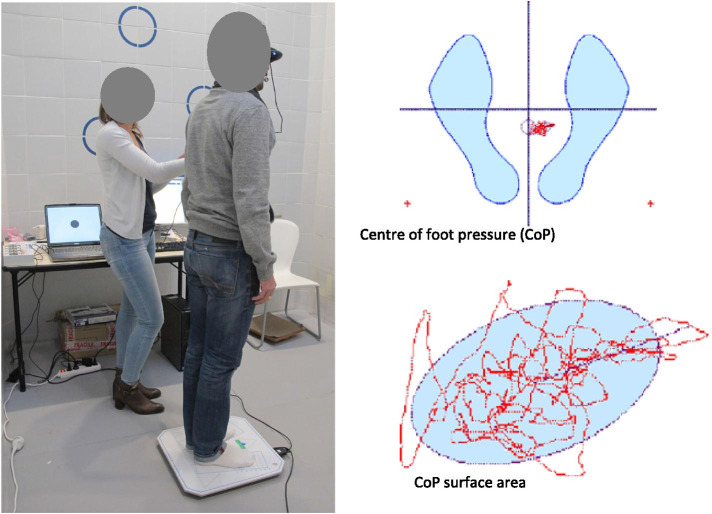
Representation of the posturography measurements used in the study. The patient stands on the posturography platform (left), and the center of foot pressure of both feet (CoP) is recorded (top right). The area covered by the CoP sway path (confidence ellipse covering 90% of the points; bottom right) is then calculated

As part of the rehabilitation program to enable patients to achieve specific goals for RTP, a posturography platform (Medicapteurs, Balma, France) [25] was used to assess postural stability at 6 months following ACLR. Adequate testing preparation was provided to the patients to ensure their familiarity and understanding of the exercise. During the final measurement the patients stood barefoot, with their legs abducted 30°, and their arms along the body, for one minute on the posturography platform, facing a visual cue on the wall. The platform recorded the center of foot pressure (CoP) and its sway path, and calculated the surface area of the ellipse that covers 90% of the data points (Fig. 1). Posturography was evaluated in 3 conditions:

- Platform stable with eyes open (PsEo,)
- Platform stable with eyes closed (PsEc)
- Platform unstable (foam surface) with eyes open (PuEo)

Two ratios were calculated to indicate the use of somatosensory and visual cues for postural control. The somatosensory ratio, calculated by dividing the surface area of the ellipse in PsEc by that in PsEo, represents the use of somatosensory input when vision is impaired. A lower somatosensory ratio indicates better neuromuscular proprioception. The visual ratio, calculated by dividing the surface area of the ellipse in PuEo by that in PsEo, represents the use of visual input when somatosensory input is impaired [25]. A lower visual ratio indicates more efficient use and/or greater reliance on visual cues to maintain balance.

### Statistical analysis

A priori sample size calculation was performed to determine whether somatosensory ratios would improve (decrease) by at least 25% following ACLR. A recent study indicated a mean preoperative somatosensory ratio of 1.46 ± 0.8 [25]. Assuming equal standard deviations, a minimum of 40 patients is required to determine a statistical significance, with a power of 0.80 and alpha of 0.05. Descriptive statistics were used to summarize the data. Shapiro–Wilk tests were used to assess the normality of distributions. Comparisons between non-parametric data were evaluated using Wilcoxon rank-sum or Mann–Whitney U tests. Univariable linear regression analyses were performed to determine associations of somatosensory and visual ratios with twelve variables (age, sex, BMI, graft type, extra-articular tenodesis, time between injury and surgery, rehabilitation protocol, and postoperative leg strength deficit, anteroposterior laxity, as well as Lysholm, IKDC and KOOS scores). Multivariable analyses were not performed due to the limited cohort size. Statistical analyses were performed using R version 3.3.2 (R Foundation for Statistical Computing, Vienna, Austria). *P*-values < 0.05 were considered statistically significant.

## Results

**Fig. 2:**
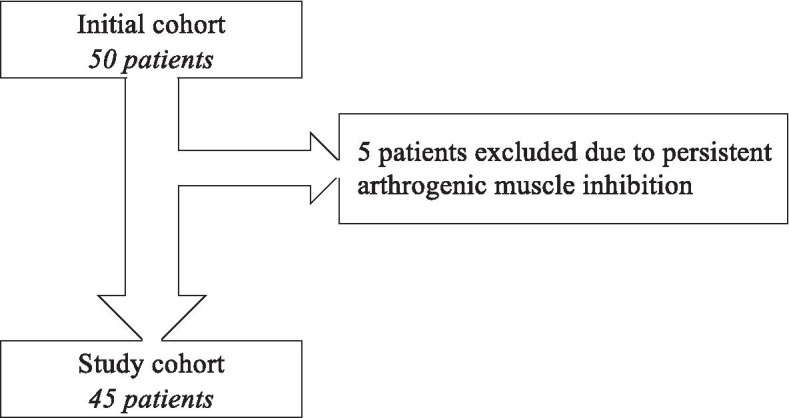
Flowchart

At a minimum follow-up of 6 months, no surgical or postoperative complication was reported; however, 5 patients were excluded due to persistent arthrogenic quadriceps muscle inhibition (Fig. 2). The remaining 45 patients comprised 34 men (76%) and 11 women (24%), aged 27 ± 6 years, with a BMI of 23 ± 3 kg/m^2^ (Table 1).

**Table 1:** Pre- and intra-operative data

	**Total cohort (*n*** = **45)**	
	**Mean ± *SD***	**(Range)**
**Sex**		
* Men*	34 (76%)	
* Women*	11 (24%)	
**Age (years)**	27.0 ± 6.2	(18 – 43)
BMI (kg/m^2^)	23.0 ± 2.7	(18 – 30)
**Time between injury and surgery (weeks)**	23.6 ± 32.6	(2 – 171)
**Graft type**		
* HT*	19 (42%)	
* BTB*	26 (58%)	
**Medial meniscal treatment**		
* None*	29 (64%)	
* Meniscectomy*	7 (16%)	
* Suture*	9 (20%)	
**Lateral meniscal treatment**		
* None*	41 (91%)	
* Meniscectomy*	4 (9%)	
* Suture*	0 (0%)	
**Extra-articular tenodesis**	22 (49%)	
**Rehabilitation protocol**		
* Land-based therapy*	21 (47%)	
* Aquatic therapy*	24 (53%)	

*HT* Hamstring autograft, *BTB* Bone-patellar-tendon-bone autograft

  The sway surface area using posturography did not change following ACLR (Table 2). In addition, the somatosensory ratio did not change, while the visual ratio decreased significantly from 5.73 ± 4.13 to 3.07 ± 1.96 (*p* = 0.002), indicating greater reliance on visual cues to maintain balance.

**Table 2:** Clinical outcomes

	**Total cohort (*n*** = **45)**				***p-value***
	***Preoperative***		***Postoperative***		
	**Mean ± *SD***	**(Range)**	**Mean ± *SD***	**(Range)**	
**Surface area (mm**^2^**)**					
* Platform stable, Eyes open (PsEo)*	152 ± 112	(47 – 782)	199 ± 180	(56 – 1087)	*0.105*
* Platform stable, Eyes closed (PsEc)*	195 ± 115	(34 – 603)	214 ± 114	(45 – 555)	*0.308*
* Platform unstable, Eyes open (PuEo)*	470 ± 246	(103 – 1459)	430 ± 166	(212 – 844)	*0.547*
**Somatosensory ratio (PsEc / PsEo)**	1.43 ± 0.71	(0.46 – 3.21)	1.39 ± 0.78	(0.27 – 3.74)	*0.967*
**Visual ratio (PuEo / PsEo)**	5.73 ± 4.13	(0.95 – 14.72)	3.07 ± 1.96	(0.58 – 9.52)	*0.002*
**Lysholm score**	66.2 ± 18.9	(19.0 – 97.0)	83.6 ± 12.8	(50.0 – 100.0)	*0.001*
**IKDC**	41.1 ± 11.4	(17.0 – 65.5)	56.8 ± 9.6	(39.0 – 79.0)	< 0.001
**KOOS**					
* Symptoms*	69.2 ± 17.0	(32.1 – 96.4)	75.6 ± 12.2	(42.9 – 92.9)	*0.098*
* Pain*	71.9 ± 15.2	(33.3 – 94.4)	83.2 ± 13.5	(47.2 – 100.0)	< 0.001
* ADL*	80.8 ± 16.1	(44.1 – 100.0)	92.8 ± 9.6	(64.7 – 100.0)	< 0.001
* Sports*	38.3 ± 22.5	(0.0 – 85.0)	68.6 ± 16.1	(35.0 – 100.0)	< 0.001
* QoL*	29.3 ± 20.4	(0.0 – 87.5)	57.8 ± 20.0	(12.5 – 100.0)	< 0.001
**Isokinetic tests**					
* Extension deficit (%)*			17.2 ± 17.5	(-12.6 – 82.9)	
* Flexion deficit (%)*			-4.8 ± 28.9	(-54.3 – 65.9)	
* Flexion/Extension (F/E) ratio*			-0.8 ± 0.24	(-2.06 – 0.47)	
**Anteroposterior laxity**					
* Absolute (ipsilateral, mm)*			5.2 ± 1.2	(3 – 8)	
* Differential (side-to-side difference, mm)*			0.7 ± 0.6	(0 – 3)	

Univariable analyses revealed that the somatosensory ratio was significantly lower for patients who performed aquatic therapy (β = -0.50; *p* = 0.045) (Table 3), but was not associated with knee laxity, muscle strength or clinical scores. The visual ratio was significantly higher in patients who received HT autografts (β = 1.32; *p* = 0.049), but was not associated with knee laxity, muscle strength or clinical scores.

**Table 3:** Univariable linear regression analyses to identify factors associated with postoperative posturography measurements

	**Somatosensory ratio**			**Visual ratio**		
	***(PsEc / PsEo)***			***(PuEc / PsEo)***		
	**β**	**C.I**	***p-value***	**β**	**C.I**	***p-value***
**Male sex**	-0.13	(-0.70 – 0.44)	*0.638*	-0.18	(-1.63 – 1.28)	*0.805*
**Age**	0.03	(-0.07 – 0.01)	*0.088*	0.03	(-0.07 – 0.13)	*0.547*
**BMI (kg/m2)**	-0.05	(-0.14 – 0.04)	*0.264*	-0.11	(-0.37 – 0.14)	*0.376*
**Graft type**						
* BTB*	REF			REF		
* HT*	0.19	(-0.35 – 0.72)	*0.478*	1.32	(0.01 – 2.64)	*0.049*
**Extra-articular tenodesis**	-0.26	(-0.78 – 0.25)	*0.305*	0.54	(-0.79 – 1.87)	*0.411*
**Time from injury to surgery** (weeks)	0.01	(-0.00 – 0.01)	*0.138*	0.00	(-0.02 – 0.01)	*0.621*
**Rehabilitation protocol**						
* Land-based therapy*	REF			REF		
* Aquatic therapy*	-0.50	(-1.00 – -0.01)	*0.045*	-0.34	(-0.99 – 1.66)	*0.610*
**Isokinetic testing**						
* Extension deficit (%)*	0.00	(-0.02 – 0.01)	*0.563*	0.00	(-0.04 – 0.04)	*0.973*
* Flexion deficit (%)*	0.01	(-0.02 – 0.00)	*0.171*	0.01	(-0.04 – 0.02)	*0.501*
* F/E ratio*	-0.08	(-1.44 – 1.28)	*0.904*	0.17	(-3.36 – 3.71)	*0.921*
**Laximetry**						
* Absolute laxity (mm)*	0.07	(-0.14 – 0.29)	*0.480*	0.21	(-0.33 – 0.75)	*0.434*
* Side-to-side difference (mm)*	0.15	(-0.27 – 0.57)	*0.474*	0.09	(-0.98 – 1.16)	*0.869*
**Lysholm**	0.03	(-0.35 – 0.40)	*0.889*	0.04	(-0.92 – 1.00)	*0.933*
**IKDC**	0.01	(-0.02 – 0.04)	*0.586*	0.01	(-0.06 – 0.08)	*0.785*
**KOOS**						
* Symptoms*	0.01	(-0.01 – 0.03)	*0.317*	0.00	(-0.06 – 0.05)	*0.895*
* Pain*	0.00	(-0.02 – 0.02)	*0.829*	0.01	(-0.06 – 0.04)	*0.815*
* ADL*	0.01	(-0.02 – 0.03)	*0.710*	0.00	(-0.07 – 0.07)	*0.929*
* Sports*	0.00	(-0.01 – 0.02)	*0.788*	0.02	(-0.06 – 0.02)	*0.404*
* QoL*	0.00	(-0.01 – 0.01)	*0.849*	0.01	(-0.03 – 0.04)	*0.715*

*β* Regression coefficient, *C.I.* 95% confidence interval

## Discussion

The most important finding of the present study was that, following ACLR, visual ratios decreased significantly, while somatosensory ratios did not change. This may suggest that there is little or no improvement in neuromuscular proprioception and therefore greater reliance on visual cues to maintain balance. Neither ratio was associated with leg strength, knee laxity and functional scores. These findings refute the hypothesis that proprioceptive feedback improves at 6 months after ACLR, irrespective of knee laxity, muscle strength and clinical scores. It is worth noting that there was little or no change in sway surface area after ACLR, indicating that at 6 postoperative months, the intervention did not improve overall patient balance. Proprioception, knee laxity and muscle strength all contribute to postural control [1] and posturography can provide useful additional information on the functional recovery of patients after ACLR. Combined with laximetry and isokinetic testing, posturography allows a holistic assessment of patient ability to return to sports. However, to the authors’ knowledge, there are no recommended balance thresholds governing return to sport after ACLR.

The finding that sway surface area does not improve after ACLR is consistent with previous studies [25], although while using a different posturography platform and protocol, 2 studies did find balance improvements at 6 and 12 months following ACLR [4, 5]. Nevertheless, patients who had ACLR demonstrate significantly greater sway surface area than control patients [4, 5, 12], suggesting that postural control is not fully restored. Furthermore, somatosensory and visual ratios are greater than those reported for control patients in a different study [9] using a similar device and protocol, suggesting that ACL tears may impair the ability of patients to use somatosensory and visual cues for balance.

In the present study, the somatosensory ratio did not change after ACLR, but the visual ratio significantly decreased. Univariable analysis revealed that patients who performed aquatic therapy had significantly lower somatosensory ratios. This improvement could be due to limited reliance on visual cues in water, which stimulates reliance on proprioceptive and/or vestibular cues. This is supported by recent findings that somatosensory and cerebellar systems have reduced activity after ACLR, while the visual and nigrostriatal systems have a stable increase in activity [4]. These observations are coherent with a model where the brain balances input from several sources, including somatosensory and visual inputs. A reduction in one input can thereby be compensated for by an increase in reliance on other inputs [2]. Thus, after ACLR, patients may become more dependent on visual input for balancing. These findings contradict older observations, that reliance on visual input during single-leg stance increases only in ACL-deficient patients, but not in patients who had ACLR [35].

In the present cohort, patients who had the addition of aquatic therapy instead of only the conventional rehabilitation protocol had lower somatosensory ratios, suggesting that the aquatic therapy may improve the recovery of somatosensory contribution to postural control [25]. By impairing visual postural correction while exercising, aquatic therapy may increase somatosensory reliance [25]. This is consistent with the recent finding that training-induced structural plasticity in brain regions associated with proprioceptive postural control were associated with improved balance in blind individuals [30]. Further, aquatic therapy was shown to provide faster recovery, allowing for an earlier return to social, sporting and professional activities [25]. Rehabilitation strategies may therefore play an important role in how patients adapt their postural control mechanisms after ACLR, who could benefit further from proprioceptive training with limitation or deprivation of visual compensation. The clinical relevance of this is that posturography can provide useful information to help research following ACLR and to predict successful RTP.

This study evaluated the utility of a common method used to assess patient postural control in evaluating outcomes of ACLR. Posturography was independent from leg strength, knee laxity and functional scores. Furthermore, this study highlighted the importance of rehabilitation for somatosensory recovery after ACLR. Its main limitation is the lack of a control group of knees without ACL tears, which prevents comparison to normal posturography measurements. In addition, posturography is a bipedal exam and therefore the unaffected leg could compensate for the decreased proprioception of the affected leg. Another limitation was the absence of preoperative laxity and isokinetic testing. Finally, the cohort was too small to evaluate the effect of meniscal lesions, or to perform multivariable analyses. Further comparative studies with larger cohorts and multiple follow-up evaluations would be beneficial to understand how proprioceptive and visual feedbacks contribute to postural control in the medium and long-terms.

## Conclusion

At 6 months following ACLR, visual ratios decreased significantly, while somatosensory ratios did not change. This suggests that there is little or no improvement in neuromuscular proprioception and therefore greater reliance on visual cues to maintain balance. The clinical relevance of this study is that posturography can provide useful information to help research following ACLR and to predict successful RTP.

## References

[1] Ageberg E, Roberts D, Holmstrom E, Friden T (2005). Balance in single-limb stance in patients with anterior cruciate ligament injury: relation to knee laxity, proprioception, muscle strength, and subjective function. Am J Sports Med.

[2] Alcock L, O’Brien TD, Vanicek N (2018). Association between somatosensory, visual and vestibular contributions to postural control, reactive balance capacity and healthy ageing in older women. Health Care Women Int.

[3] Angoules AG, Mavrogenis AF, Dimitriou R, Karzis K, Drakoulakis E, Michos J, Papagelopoulos PJ (2011). Knee proprioception following ACL reconstruction; a prospective trial comparing hamstrings with bone-patellar tendon-bone autograft. Knee.

[4] Bartels T, Brehme K, Pyschik M, Pollak R, Schaffrath N, Schulze S, Delank KS, Laudner K, Schwesig R (2019). Postural stability and regulation before and after anterior cruciate ligament reconstruction - a two years longitudinal study. Phys Ther Sport.

[5] Bartels T, Brehme K, Pyschik M, Schulze S, Delank KS, Fieseler G, Laudner KG, Hermassi S, Schwesig R (2018). Pre- and postoperative postural regulation following anterior cruciate ligament reconstruction. J Exerc Rehabil.

[6] Boerboom AL, Huizinga MR, Kaan WA, Stewart RE, Hof AL, Bulstra SK, Diercks RL (2008). Validation of a method to measure the proprioception of the knee. Gait Posture.

[7] Bouguennec N, Odri GA, Graveleau N, Colombet P (2015). Comparative reproducibility of TELOS and GNRB(R) for instrumental measurement of anterior tibial translation in normal knees. Orthop Traumatol Surg Res.

[8] Clark RA, Howells B, Feller J, Whitehead T, Webster KE (2014). Clinic-based assessment of weight-bearing asymmetry during squatting in people with anterior cruciate ligament reconstruction using Nintendo Wii Balance Boards. Arch Phys Med Rehabil.

[9] Colnat-Coulbois S, Gauchard GC, Maillard L, Vignal JP, Vespignani H, Auque J, Perrin PP (2011). Drug-resistant temporal lobe epilepsy is associated with postural control abnormalities. Epilepsy Behav.

[10] Cronstrom A (2018). Is poor proprioception associated with worse movement quality of the knee in individuals with anterior cruciate ligament deficiency or reconstruction?. J Phys Ther Sci.

[11] Culvenor AG, Alexander BC, Clark RA, Collins NJ, Ageberg E, Morris HG, Whitehead TS, Crossley KM (2016). Dynamic single-leg postural control is impaired bilaterally following anterior cruciate ligament reconstruction: implications for reinjury risk. J Orthop Sports Phys Ther.

[12] Dauty M, Collon S, Dubois C (2010). Change in posture control after recent knee anterior cruciate ligament reconstruction?. Clin Physiol Funct Imaging.

[13] Dejour D, Pungitore M, Valluy J, Nover L, Saffarini M, Demey G (2019). Preoperative laxity in ACL-deficient knees increases with posterior tibial slope and medial meniscal tears. Knee Surg Sports Traumatol Arthrosc.

[14] Domingues PC, Serenza FS, Muniz TB, de Oliveira LFL, Salim R, Fogagnolo F, Kfuri M, Ferreira AM (2018). The relationship between performance on the modified star excursion balance test and the knee muscle strength before and after anterior cruciate ligament reconstruction. Knee.

[15] Fulton J, Wright K, Kelly M, Zebrosky B, Zanis M, Drvol C, Butler R (2014). Injury risk is altered by previous injury: a systematic review of the literature and presentation of causative neuromuscular factors. Int J Sports Phys Ther.

[16] Hefti F, Muller W, Jakob RP, Staubli HU (1993). Evaluation of knee ligament injuries with the IKDC form. Knee Surg Sports Traumatol Arthrosc.

[17] Hetsroni I, Wiener Y, Ben-Sira D, Iacono AD, Marom N, van Stee M, Ayalon M (2020). Symmetries in muscle torque and landing kinematics are associated with maintenance of sports participation at 5 to 10 years after ACL reconstruction in young men. Orthop J Sports Med.

[18] Jenny JY, Puliero B, Schockmel G, Harnoist S, Clavert P (2017). Experimental validation of the GNRB((R)) for measuring anterior tibial translation. Orthop Traumatol Surg Res.

[19] Kim HJ, Lee JH, Lee DH (2017). Proprioception in patients with anterior cruciate ligament tears: a meta-analysis comparing injured and uninjured limbs. Am J Sports Med.

[20] Lee HM, Cheng CK, Liau JJ (2009). Correlation between proprioception, muscle strength, knee laxity, and dynamic standing balance in patients with chronic anterior cruciate ligament deficiency. Knee.

[21] Lim JM, Cho JJ, Kim TY, Yoon BC (2019). Isokinetic knee strength and proprioception before and after anterior cruciate ligament reconstruction: a comparison between home-based and supervised rehabilitation. J Back Musculoskelet Rehabil.

[22] Magnussen RA, Duthon V, Servien E, Neyret P (2013). Anterior cruciate ligament reconstruction and osteoarthritis: evidence from long-term follow-up and potential solutions. Cartilage.

[23] Parus K, Lisinski P, Huber J (2015). Body balance control deficiencies following ACL reconstruction combined with medial meniscus suture. A preliminary report. Orthop Traumatol Surg Res.

[24] Paterno MV, Schmitt LC, Ford KR, Rauh MJ, Myer GD, Huang B, Hewett TE (2010). Biomechanical measures during landing and postural stability predict second anterior cruciate ligament injury after anterior cruciate ligament reconstruction and return to sport. Am J Sports Med.

[25] Peultier-Celli L, Mainard D, Wein F, Paris N, Boisseau P, Ferry A, Gueguen R, Chary-Valckenaere I, Paysant J, Perrin P (2017). Comparison of an innovative rehabilitation, combining reduced conventional rehabilitation with aquatic therapy, and a conventional rehabilitation after anterior cruciate ligament reconstruction in athletes. Front Surg.

[26] Reider B, Arcand MA, Diehl LH, Mroczek K, Abulencia A, Stroud CC, Palm M, Gilbertson J, Staszak P (2003). Proprioception of the knee before and after anterior cruciate ligament reconstruction. Arthroscopy.

[27] Relph N, Herrington L, Tyson S (2014). The effects of ACL injury on knee proprioception: a meta-analysis. Physiotherapy.

[28] Roberts D, Ageberg E, Andersson G, Friden T (2007). Clinical measurements of proprioception, muscle strength and laxity in relation to function in the ACL-injured knee. Knee Surg Sports Traumatol Arthrosc.

[29] Rochcongar G, Cucurulo T, Ameline T, Potel JF, Dalmay F, Pujol N, Salle de Chou E, Lutz C, Ehkirch FP, Le Henaff G, Laporte C, Seil R, Gunepin FX, Sonnery-Cottet B, la SFA (2015). Meniscal survival rate after anterior cruciate ligament reconstruction. Orthop Traumatol Surg Res.

[30] Rogge AK, Hotting K, Nagel V, Zech A, Holig C, Roder B (2019). Improved balance performance accompanied by structural plasticity in blind adults after training. Neuropsychologia.

[31] Roos EM, Roos HP, Lohmander LS, Ekdahl C, Beynnon BD (1998). Knee Injury and Osteoarthritis Outcome Score (KOOS)–development of a self-administered outcome measure. J Orthop Sports Phys Ther.

[32] Samitier G, Marcano AI, Alentorn-Geli E, Cugat R, Farmer KW, Moser MW (2015). Failure of anterior cruciate ligament reconstruction. Arch Bone JtSurg.

[33] San Martin-Mohr C, Cristi-Sanchez I, Pincheira PA, Reyes A, Berral FJ, Oyarzo C (2018). Knee sensorimotor control following anterior cruciate ligament reconstruction: a comparison between reconstruction techniques. PLoS ONE.

[34] Tegner Y, Lysholm J (1985). Rating systems in the evaluation of knee ligament injuries. Clin Orthop Relat Res.

[35] Wikstrom EA, Song K, Pietrosimone BG, Blackburn JT, Padua DA (2017). Visual utilization during postural control in anterior cruciate ligament- deficient and -reconstructed patients: systematic reviews and meta-analyses. Arch Phys Med Rehabil.

[36] Wright RW, Dunn WR, Amendola A, Andrish JT, Flanigan DC, Jones M, Kaeding CC, Marx RG, Matava MJ, McCarty EC, Parker RD, Vidal A, Wolcott M, Wolf BR, Spindler KP, Cohort M (2007). Anterior cruciate ligament revision reconstruction: two-year results from the MOON cohort. J Knee Surg.

